# Design and immunogenicity of a quadrivalent mRNA vaccine targeting HSV-2 with comparative evaluation of co-formulated and admixed formulations

**DOI:** 10.3389/fimmu.2025.1712691

**Published:** 2025-12-05

**Authors:** Youngran Cho, Chanwoo Lee, Sang-In Park, Yeongjun Kim, Yu-Sun Lee, Seonghyun Lee, Subin Yoon, Gahyun Roh, Dahyeon Ha, Ayoung Oh, Kyusang Cho, Jisun Lee, Hyo-Jung Park, Hye-Ra Lee, Jae-Hwan Nam

**Affiliations:** 1 Department of Medical and Biological Sciences, The Catholic University of Korea, Bucheon, Republic of Korea; 2 Brain Korea 21 Four (BK21) Four Department of Biotechnology, The Catholic University of Korea, Bucheon, Republic of Korea; 3 Department of Biotechnology and Bioinformatics, College of Science and Technology, Korea University, Sejong, Republic of Korea; 4 Department of Biomedical Laboratory Science, Daegu Haany University, Gyeongsan, Republic of Korea; 5 Department of Lab Medicine, College of Medicine, Korea University, Seoul, Republic of Korea

**Keywords:** herpes simplex virus 2, mRNA vaccine, quadrivalent vaccine, long-term immunity, genital herpes

## Abstract

**Introduction:**

The globally prevalent herpes simplex virus 2 (HSV-2) establishes lifelong latent infections in sensory neurons and causes recurrent genital disease. However, no vaccine is available to prevent HSV-2 infection. The mRNA vaccine platform offers distinct advantages over protein-based approaches, including rapid antigen design, scalable production, and efficient intracellular expression.

**Methods:**

A prophylactic quadrivalent mRNA vaccine encoding full-length HSV-2 glycoprotein B (gB2), C (gC2), D (gD2), and E (gE2) was developed. Its immunogenicity and protective efficacy were evaluated in a murine intravaginal challenge model.

**Results:**

Quadrivalent mRNA vaccine-immunized mice showed robust HSV-2-specific immune responses, including high titers of neutralizing antibodies and strong T cell responses, which persisted for at least 16 weeks. Upon viral challenge, vaccinated animals were fully protected from genital disease and exhibited significantly reduced viral copy numbers in the genital tract. Vaccination also inhibited the establishment of latent infections in the dorsal root ganglia, as evidenced by markedly lower HSV-2 DNA levels than those in mock-vaccinated controls. Comparative analysis showed no significant difference between co-formulated and admixed lipid nanoparticle formulations, indicating flexibility in vaccine manufacturing without compromising efficacy.

**Discussion:**

The quadrivalent mRNA vaccine provides strong and durable protection against both primary and latent infection, supporting its potential as a promising candidate for the prevention of genital herpes.

## 1 Introduction

Herpes simplex virus 2 (HSV-2) is a globally prevalent sexually transmitted virus, with an estimated seroprevalence of 13% among individuals aged 15–49 (1). Following primary mucocutaneous infection, HSV-2 establishes lifelong latency in sensory neurons and periodically reactivates, leading to recurrent genital lesions or asymptomatic viral shedding that collectively maintain viral transmission. HSV-2 is primarily spread through sexual contact but can also be transmitted perinatally during vaginal delivery. Globally, neonatal herpes affects approximately 14,000 infants annually and is associated with high morbidity and mortality (2). Moreover, HSV-2 infection significantly increases the risk of acquiring and transmitting human immunodeficiency virus (HIV) with a two- to five-fold increase in susceptibility (3).

Although the HSV-2 infection remains a significant global health concern, developing a prophylactic vaccine remains challenging. Previous vaccine strategies have primarily targeted the viral envelope glycoproteins critical for host cell entry, particularly the HSV-2 glycoproteins B (gB2) and D (gD2). gB2 mediates initial attachment to host cells through interactions with heparan sulfate proteoglycans (HSPGs), whereas gD2 binds to host receptors, such as Nectin-1 and herpesvirus entry mediator (HVEM) (4). This receptor engagement induces conformational changes that enable gB2 to coordinate with gH/gL to mediate fusion of the viral envelope with the host membrane (4–6). Given their essential roles in viral entry, gB2 and gD2 are central components in the subunit vaccine design. A clinical trial evaluating a gB2/gD2-based vaccine showed a delay in infection onset, but ultimately failed to achieve durable protection (7). A more recent trivalent subunit vaccine incorporating gC2, gD2, and gE2 demonstrated promising immunogenicity in nonhuman primates and conferred protection against genital lesions in guinea pig models, indicating its potential for further clinical development (8). Furthermore, a trivalent mRNA vaccine encoding gC2, gD2, and gE2 showed improved efficacy compared to its protein subunit counterpart. This mRNA-based vaccine elicited robust humoral and CD4^+^ T cell responses and provided superior protection against subclinical infections, as reflected by the viral loads in mucosal and neural tissues in the murine model and vaginal shedding in the guinea pig model (9).

In this study, we evaluated a quadrivalent mRNA vaccine encoding HSV-2 glycoproteins gB2, gC2, gD2, and gE2. Therefore, we analyzed the immunological characteristics of each gene individually. The results showed that the gB2, gC2, and gD2 mRNA vaccines effectively induced antibodies and CD4^+^ activation, whereas the gE2 mRNA vaccine induced CD8^+^ T cells but failed to induce antibodies, demonstrating distinct immunological characteristics. Based on these results, the quadrivalent mRNA vaccine, which contained all four genes, induced strong and sustained immune responses during the early stages of immunization, including robust CD4^+^ responses and increased numbers of activated CD8^+^ effector T cells. These findings may contribute to the development of strategies for improving the efficacy and durability of HSV-2 vaccines.

## 2 Materials and methods

### 2.1 mRNA production

gB2, gC2, gD2, and gE2 mRNAs were generated from DNA templates encoding full-length gB2 (GenBank accession no. YP_009137179.1), gC2 (GenBank accession no. WZW64493.1), gD2 (GenBank accession no. AAA45841.1), and gE2 (GenBank accession no. YP_009137220.1). DNA sequences were synthesized by Azenta Life Sciences (Burlington, MA, USA) and cloned into the mRNA platform backbone plasmid (T7 promoter – 5′ UTR – multiple cloning sites – 3′ UTR – poly(A) tail) (10). mRNAs were transcribed from the DNA templates using the EZ T7 High Yield *In Vitro* Transcription Kit (Enzynomics, Daejeon, Republic of Korea) with the SC101 Cap 1 analog (ST Pharm, Siheung, Republic of Korea), and uridine triphosphate (UTP) was replaced with N1-Methylpseudo-UTP (TriLink BioTechnologies, San Diego, CA, USA). Transcribed mRNAs were precipitated with lithium chloride and further purified using a cellulose-based method (11). RNA concentration and purity ratios were determined using a NanoDrop 2000 spectrophotometer (Thermo Fisher Scientific, Waltham, MA, USA) and RNA integrity was confirmed by denaturing agarose gel electrophoresis.

### 2.2 mRNA–LNP formulation

Lipid nanoparticle (LNP) components, including SM-102, 1,2-distearoyl-sn-glycero-3-phosphocholine (DSPC), cholesterol, and 1,2-dimyristoyl-rac-glycero-3-methoxypolyethylene glycol-2000 (DMG-PEG 2000), were dissolved in ethanol at a molar ratio of 50:10:38.5:1.5. mRNAs were prepared in 50 mM citrate buffer (pH 4) at an N/P ratio of 6. mRNAs were encapsulated in LNPs by microfluidically mixing the aqueous and organic phases in a 3:1 ratio using an enCELL-Master (enParticle, Busan, Republic of Korea). For the co-formulation, gB2, gC2, gD2, and gE2 mRNAs were combined in equal amounts before being encapsulated in LNPs. For the admixed formulation, equal amounts of gB2, gC2, gD2, and gE2 mRNAs were separately encapsulated in LNPs and combined. mRNA–LNPs were buffer-exchanged into phosphate-buffered saline (PBS) and concentrated using an Amicon Ultra-15 Centrifugal Filter at 30 K. The size of the mRNA-loaded LNPs was measured using a Zetasizer Nano ZS (Malvern Panalytical Ltd., Malvern, UK).

### 2.3 Mice

The animal studies were approved by the Institutional Animal Care and Use Committee of the Catholic University of Korea (approval no. CUK-IACUC-2023-045). Female BALB/c mice, aged 5–6 weeks, were obtained from Koatech (Pyeongtaek, Republic of Korea) and housed under specific pathogen-free conditions with a standard light/dark cycle, 50–60% humidity, and a temperature of 21–25°C. All the experimental procedures complied with the guidelines of the Institutional Animal Care and Use Committee of the Catholic University of Korea. Mice were anesthetized by inhalation of 3–5% isoflurane in oxygen at a flow rate of 1–2 L/min for induction, and 1–3% isoflurane in oxygen at a flow rate of 1 L/min for maintenance. Mice were euthanized by gradual exposure to CO_2_ at a flow rate of 30–70% of chamber volume per minute until cessation of respiration, in accordance with institutional animal guidelines.

### 2.4 Immunizations

In all the experiments, mRNA vaccines were injected twice into the hind gastrocnemius muscle of 6-9-week-old female mice at a two-week interval. Mice received a prime immunization at week 0, followed by a booster immunization at week 2. The standard immunization dose was 10 μg of mRNA per antigen. For the dose-dependent immunogenicity study, mice received doses of 1 μg, 5 μg, or 10 μg of mRNA per antigen.

### 2.5 IgG ELISA

Total IgG, IgG1, and IgG2a titers in serum were determined by ELISA. High-binding 96-well plates were coated with 250 ng/well of HSV-2 strain MS lysates in carbonate coating buffer (pH 9.5) at 4°C overnight and blocked with 1% bovine serum albumin (BSA) in PBS for 1 h at room temperature. Two-fold serial dilutions of serum in blocking buffer were added to the plates and incubated for 2 h at room temperature. Colorimetric detection was performed by incubating for 1 h with horseradish peroxidase (HRP)-conjugated antibodies: anti-mouse IgG (Bethyl Laboratories, Montgomery, TX, USA) at a 1:3,000 dilution, IgG1 (Bethyl Laboratories) at a 1:3,000 dilution, or IgG2a (Bio-Rad, Hercules, CA, USA) at a 1:2,000 dilution, followed by incubation with 3,3′,5,5′-tetramethylbenzidine (TMB) substrates. The reaction was stopped with 2N H_2_SO_4_, and the absorbance was measured at 450 nm. Endpoint titers were defined as the serum dilution factor at which the absorbance value was at least two-fold greater than that of the PBS group and not less than 0.1.

### 2.6 Plaque reduction neutralization test for neutralizing antibody assay

Serum samples were serially diluted two-fold, starting at a 1:10 dilution in serum-free medium. Diluted sera were incubated with 100 plaque-forming units (PFU) of HSV-2 strain MS (Korea Bank for Pathogenic Viruses, Seoul, Republic of Korea) for 1 h at 37°C. Vero cells (Korean Cell Line Bank, Seoul, Republic of Korea) were seeded the day before infection (1×10^5^ cells/well in 24-well plates or 5×10^4^ cells/well in 96-well plates), and infected with virus-serum mixtures for 1 h at 37°C, 5% CO_2_, with gentle agitation. After adsorption, the inoculum was removed, and cells were overlaid with carboxymethylcellulose medium. Following incubation for 48 h at 37°C with 5% CO_2_, cells were fixed with 4% paraformaldehyde and stained with crystal violet solution. Plaques were counted and neutralizing antibody titers were determined as the highest serum dilution that reduced the plaque count by 50% (PRNT_50_).

### 2.7 Challenge and scoring

Mice were pretreated with medroxyprogesterone 5 days before intravaginal challenge with 1×10^5^ PFU of HSV-2 strain MS. Following infection, mice were monitored daily for weight loss and survival and evaluated every two days for genital disease and clinical signs. Genital disease was scored 1 point each for redness/erythema, hair loss, and urinary staining, with a maximum score of 3. Clinical signs were scored on a scale of 0–4: 0, no visible signs of disease; 1, slight ruffling of the fur; 2, ruffled fur, reduced mobility; 3, ruffled fur, minimal mobility; and 4, ruffled fur, minimal mobility, huddled appearance, and hind limb weakness. Mice were euthanized if they lost more than 20% of their initial body weight and exhibited genital disease symptoms.

### 2.8 Plaque assay

HSV-2 titer was determined by plaque assay. Vaginal swab samples and HSV-2 strain MS were serially diluted 10-fold. Vero cells were cultured in 96-well plates and infected with the diluted viruses or vaginal swab samples. After 1 h of incubation at 37°C with 5% CO_2_, the medium was replaced with an overlay medium containing 1% carboxymethylcellulose. Following 48 h incubation, the cells were fixed and stained with a 0.05% crystal violet solution containing methanol and formaldehyde. Viral plaques were counted after overnight staining.

### 2.9 Real-time PCR

Total genomic DNA was extracted from HSV-2-infected mouse dorsal root ganglia (DRG) using the DNeasy Blood and Tissue Kit (Qiagen, Germantown, MD, USA) following the manufacturer’s instructions. Real-time PCR was performed using SYBR Green and the CFX96 Real-Time System (Bio-Rad, Hercules, CA, USA). The primers targeting the HSV-2 US9 gene were as follows: Forward: 5′-GGCAGAAGCCTACTACTCGGAAA-3′; Reverse: 5′-CCATGCGCACGAGGAAGT-3′.

### 2.10 Histopathological analysis

Sectioned tissues from the experimental animals were submerged in 10% neutral buffered formalin, dehydrated, paraffin-embedded, and sectioned at 4-µm thickness for histological examination. Histopathological images were obtained and evaluated using an Aperio ImageScope version 12.6 (Leica Biosystems Pathology Imaging, Buffalo Grove, IL, USA). The severity of the histological changes was determined using a 5-point score system as follows: 0, no abnormality detected (NAD); 1, minimal; 2, mild; 3, moderate; 4, moderately severe; and 5, severe. The distribution was recorded as focal, multifocal, or diffuse. Recruitment of inflammatory cells and morphological alterations in the tissues were assessed by hematoxylin and eosin (H&E) staining.

### 2.11 Peptides for the stimulation of HSV-2-specific immune responses

The gB2, gC2, and gD2 peptide pools (JPT Peptide Technologies, Berlin, Germany) consisted of 15-mer peptides spanning the entire antigen sequence with an 11-amino acid overlap. The gE2 peptides consisted of five 9-mer peptides that were predicted for MHC-I binding using the NetMHCpan-4.1b server and synthesized by Peptron, Inc. (Daejeon, Republic of Korea). Splenocytes were stimulated with the gB2, gC2, or gD2 peptide pools at a final concentration of 0.7–1 μg/mL per peptide. For gE2 peptides, a total concentration of 10 μg/mL was used for the ELISPOT assay, and 25 μg/mL for the flow cytometric analysis of intracellular cytokine staining (ICS).

### 2.12 ELISPOT assay

Splenocytes secreting IFN-γ or IL-4 were measured using the ELISpot Flex (ALP) kit (Mabtech AB, Nacka, Sweden) according to the manufacturer’s protocol. The day before splenocyte harvest, 96-well filter plates were coated with capture antibodies (anti-mouse IFN-γ: clone AN18; anti-mouse IL-4: clone 11B11) at 4°C overnight and blocked with Roswell Park Memorial Institute (RPMI) medium containing 10% FBS. Subsequently, 5×10^5^ splenocytes were added to the coated plates and stimulated with antigens (gB2 peptide pool, gC2 peptide pool, gD2 peptide pool, or gE2 peptides) for 48 h at 37°C with 5% CO_2_. Biotinylated antibodies (anti-mouse IFN-γ: clone R4-6A2; anti-mouse IL-4: clone BVD6-24G2) were then added and incubated for 2 h at room temperature. Streptavidin-alkaline phosphatase (ALP) was linked to biotinylated antibodies during a 1-h incubation. Bromochloroindolyl phosphate/nitro blue tetrazolium (BCIP/NBT) substrate was added for color development, and the reaction was stopped by washing with tap water. The number of spots was counted using an AID ELISpot Reader with software 7.0 (AID GmbH, Strassberg, Germany).

### 2.13 Flow cytometry

1×10^6^ splenocytes were plated in 96-well plates and blocked with CD16/CD32 (clone 93) for 30 min at 4°C. After blocking, cells were stained with LIVE/DEAD Fixable Aqua Stain, CD8 BV605 (clone 53-6.7), CD44 FITC (clone NIM-R8), CD62L PE-Cy7 (clone MEL-14), CD127 APC (clone SB/199), and KLRG1 PE (clone 2F1/KLRG1) for 1 h at 4°C. Stained cells were then fixed with 4% paraformaldehyde and analyzed using a CytoFLEX flow cytometer with CytExpert software 2.4 (Beckman Coulter, Brea, CA, USA).

### 2.14 Flow cytometric analysis of ICS

1×10^6^ splenocytes were stimulated with antigens (gB2 peptide pool, gC2 peptide pool, gD2 peptide pool, or gE2 peptides) in RPMI medium supplemented with 10% FBS and 1% antibiotics. Brefeldin A was added 1 h post-stimulation, followed by a 12-h incubation at 37°C with 5% CO_2_. Following stimulation, cells were blocked with CD16/CD32 (clone 93) and subsequently stained with LIVE/DEAD Fixable Aqua Stain, CD4 APC-Cy7 (clone GK1.5), and CD8 BV605 (clone 53-6.7) for surface marker analysis. Cells were then permeabilized with Cytofix/Cytoperm for 30 min at 4°C and intracellularly labeled with IFN-γ APC (clone XMG1.2) or IFN-γ PE-Cy7 (clone XMG1.2), TNF-α FITC (clone MP6-XT22), and Granzyme B PE (clone QA16A02) for 1 h at 4°C. Cytokine-labeled cells were analyzed using a CytoFLEX flow cytometer with CytExpert software 2.4 (Beckman Coulter, Brea, CA, USA).

### 2.15 Statistical analysis

Graphs and statistical analyses were performed using GraphPad Prism (version 9.1.0). The Kruskal-Wallis test was used for multiple group comparisons of antibody titers, viral titers, and viral DNA copies. Dunn’s multiple comparisons test was used as a *post hoc* analysis to determine adjusted *p*-values. A two-tailed Mann-Whitney U test was used when only two groups were present. Changes in antibody titers between the two time points within the same group were analyzed using a two-tailed Wilcoxon signed-rank test. One-way analysis of variance (ANOVA) was used for multiple group comparisons of CD8^+^ T cell subsets and vaginal histopathology. Tukey’s multiple comparisons test was used for *post hoc* analysis to determine the adjusted *p*-values. T cell and splenocyte responses to HSV-2 peptide stimulation were analyzed using two-way ANOVA with Tukey’s multiple comparisons test. Two-way ANOVA with Sidak’s multiple comparisons test was used when only two groups were present. *P*-values are denoted as follows: *p < 0.05, **p < 0.01, ***p < 0.001, ****p < 0.0001, ns, not significant. Error bars represent 95% confidence intervals (CI) for antibody titers, viral titers, and viral DNA copies, and standard deviations (SD) for cellular immune responses, weight loss, genital disease, clinical signs, and vaginal histopathology.

## 3 Results

### 3.1 Immunogenicity of gB2, gC2, gD2, and gE2 mRNAs was assessed based on their capacity to induce antibody and T cell responses

**Figure 1:**
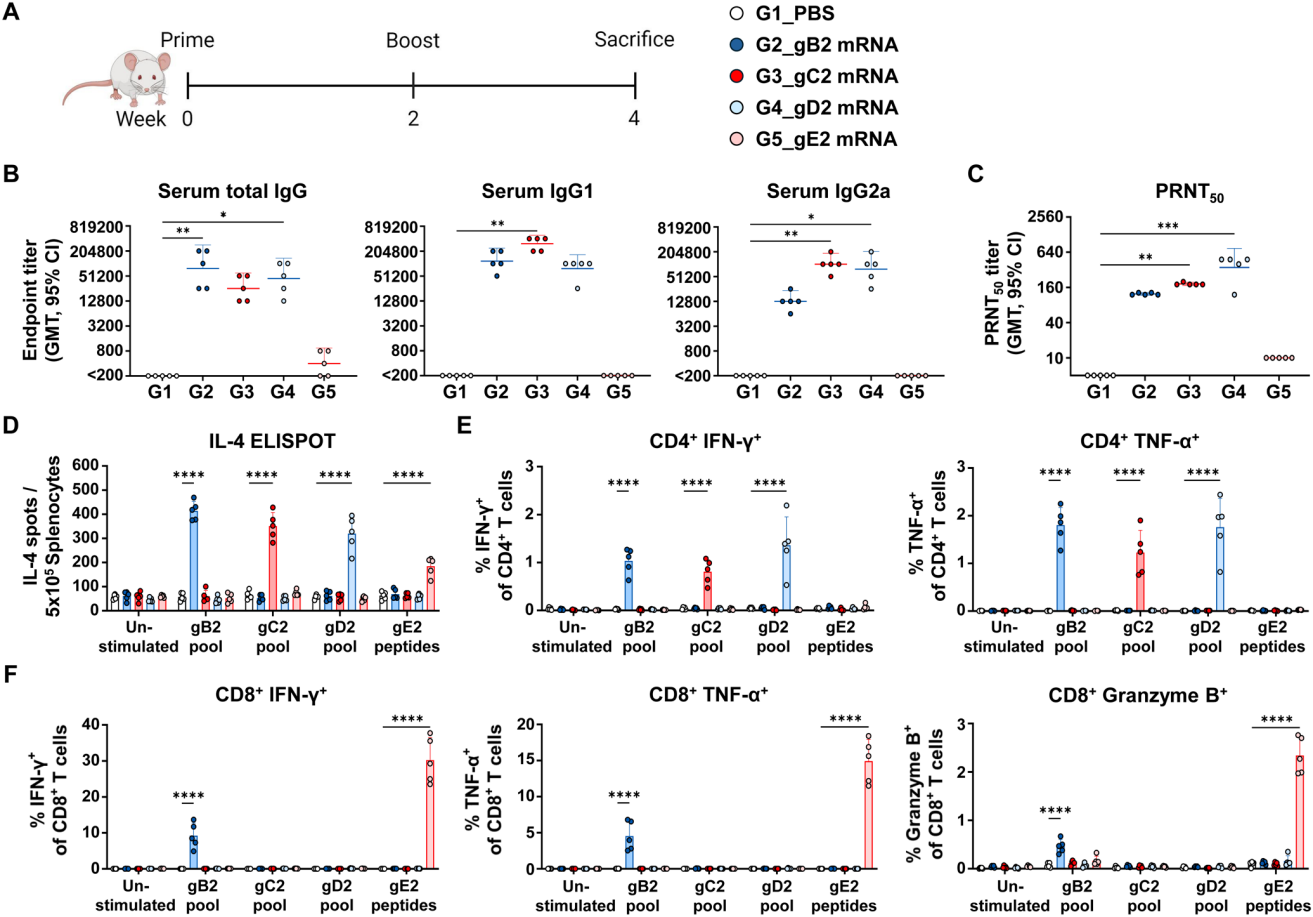
Characterization of the immunogenicity of gB2, gC2, gD2, and gE2 mRNAs. Mice were immunized twice intramuscularly with PBS or 10 μg of gB2, gC2, gD2, or gE2 mRNA. **(A)** Immunization groups and schedule. **(B)** Serum total IgG, IgG1, and IgG2a were measured using ELISA. **(C)** PRNT_50_ neutralizing antibody titers were measured using PRNT. **(D)** An IL-4 ELISPOT assay was performed on splenocytes. **(E, F)** CD4^+^ T cells producing IFN-γ or TNF-α **(E)** and CD8^+^ T cells producing IFN-γ, TNF-α, or Granzyme B **(F)** in splenocytes were analyzed using ICS/flow cytometry. Splenocytes were stimulated with the peptide pools of gB2, gC2, gD2, or the gE2 peptides prior to the ELISPOT and ICS/flow cytometry assays. Experiments involved five mice per group (n = 5/group). *P-*values were calculated using the Kruskal-Wallis test with Dunn’s multiple comparisons test **(B, C)** and two-way ANOVA with Tukey’s multiple comparisons test **(D–F)**. *p < 0.05, **p < 0.01, ***p < 0.001, ****p < 0.0001. Error bars represent 95% confidence intervals of the geometric means **(B, C)** and standard deviations of the means **(D–F)**. GMT, geometric mean titer; ELISA, enzyme-linked immunosorbent assay; PRNT, plaque reduction neutralization test; ELISPOT, enzyme-linked immunospot; ICS, intracellular cytokine staining.

To evaluate the humoral and cellular immune responses induced by gB2, gC2, gD2, and gE2 mRNAs, mice were immunized twice at a two-week interval with 10 μg of each mRNA, and sera and spleens were collected two weeks later (**Figure 1A**). Antibody analysis showed that gB2, gC2, and gD2 mRNA elicited high titers of total IgG, IgG1, and IgG2a antibodies that bound to the HSV-2 lysate, indicating strong antigen recognition. However, gE2 mRNA induced low levels of total IgG (**Figure 1B**) and neutralizing antibody titers (**Figure 1C**), suggesting a weaker humoral response. These results indicated that gB2, gC2, and gD2 contribute more effectively to viral neutralization and entry blockade.

Cellular immune responses were assessed in peptide-stimulated splenocytes. All four glycoprotein mRNAs induced significantly more IL-4-secreting cells than PBS did, although gE2 elicited weaker T-helper type 2 (Th2) responses (**Figure 1D**). T-helper type 1 (Th1) responses were evaluated by measuring cytokine-producing CD4^+^ and CD8^+^ T cells (12–14). CD4^+^ T cells producing IFN-γ and TNF-α were detected in the gB2, gC2, and gD2 groups, while CD8^+^ T cells producing IFN-γ, TNF-α, and Granzyme B were observed in the gB2 and gE2 groups (**Figures 1E, F**). Although the frequency of IFN-γ^+^ or TNF-α^+^ CD4^+^ T cells appeared modest in absolute terms, it represented a multi-fold increase relative to unstimulated controls, with high statistical significance (*p* < 0.0001). Such fold increases were generally considered highly biologically significant (8, 9, 15–18).

Additionally, the gB2 and gE2 groups showed increased frequencies of CD8^+^ T short-lived effector cells (SLEC) and effector memory T cells (TEM). These CD8^+^ TEM populations, though not antigen-specific ones, are likely to support long-term protection, given their role in controlling viral reactivation (**Supplementary Figure S1**). Overall, gB2, gC2, and gD2 mRNA elicited stronger Th2 responses and higher antibody levels than gE2 mRNA, suggesting more effective B cell activation. T cell analysis showed that gC2 and gD2 induced CD4^+^ Th1 responses, whereas gE2 primarily induced CD8^+^ type 1 cytotoxic T (Tc1) responses. Although CD4^+^ T cell responses to gE2 could not be fully assessed owing to limitations in peptide selection, their presence cannot be ruled out. Notably, gB2 mRNA induced both CD4^+^ and CD8^+^ T cell responses, reflecting its ability to generate broad cellular immunity.

### 3.2 IgG and neutralizing antibody responses to quadrivalent mRNA vaccines with two different formulations

**Figure 2:**
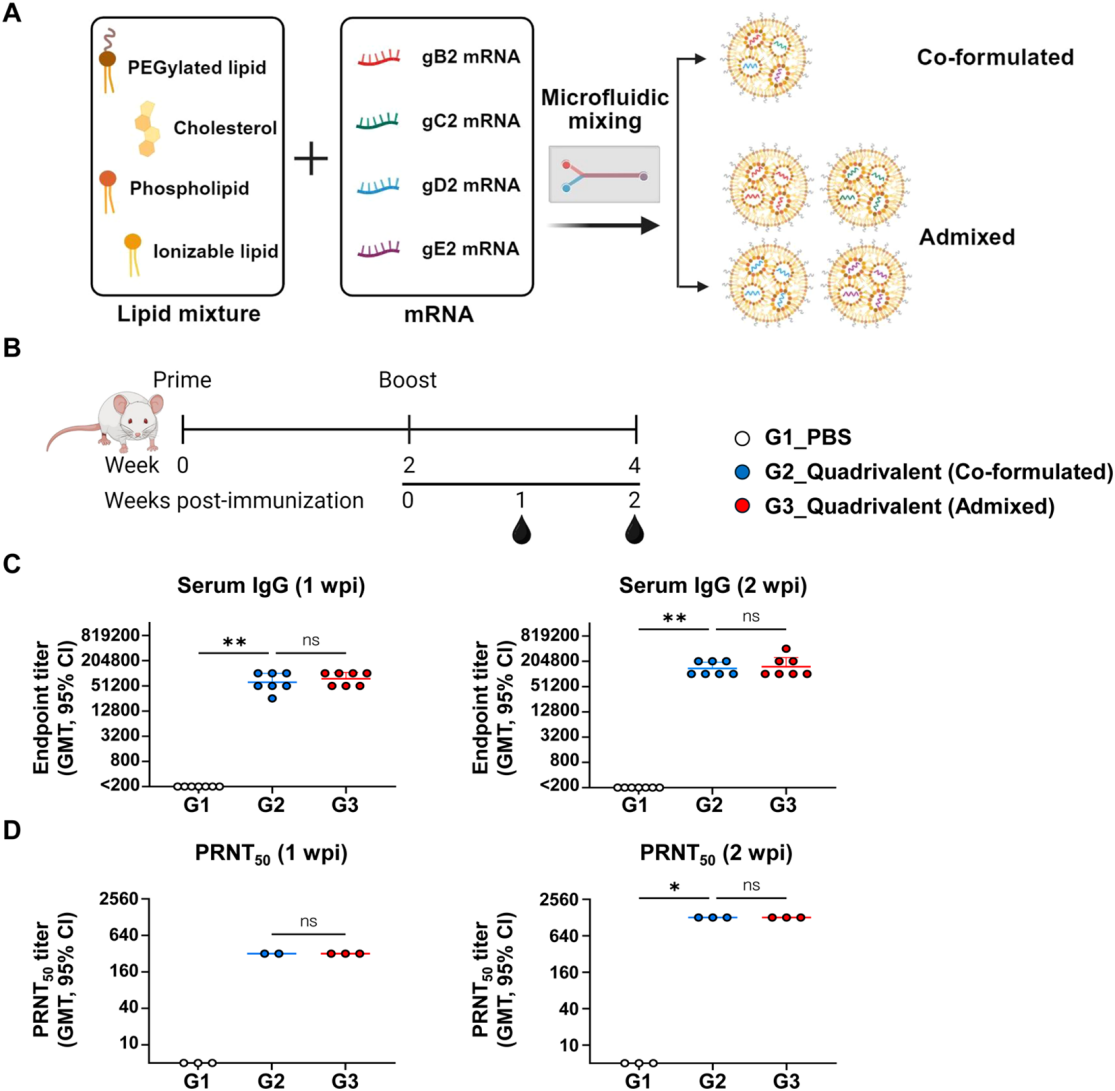
Comparison of IgG and neutralizing antibody responses between co-formulated and admixed quadrivalent mRNA vaccines. Mice were immunized twice intramuscularly with PBS, co-formulated quadrivalent mRNA vaccine, or admixed quadrivalent mRNA vaccine 4 weeks prior to HSV-2 challenge. 10 μg of mRNA per antigen was used for each group. **(A)** Schematic representation of the mRNA–LNP formulation. The quadrivalent vaccine, consisting of gB2, gC2, gD2, and gE2 mRNAs, was either co-formulated into a single LNP or formulated for each antigen and then admixed. **(B)** Immunization groups and schedule. **(C, D)** Serum IgG titers **(C)** and PRNT_50_ neutralizing antibody titers **(D)** were measured using ELISA and PRNT, respectively, on weeks 1 and 2 post-second immunization. Sera for the PRNT were pooled and tested in triplicate. For the co-formulated group (1 wpi), data were analyzed in duplicate; one replicate was excluded due to edge-localized plaque formation. Experiments involved seven mice per group (n = 7/group). *P-*values were calculated using the Kruskal-Wallis test with Dunn’s multiple comparisons test. *p < 0.05, **p < 0.01, ns, not significant. Error bars represent 95% confidence intervals of the geometric means. LNP, lipid nanoparticle; GMT, geometric mean titer; wpi, weeks post-second immunization.

The humoral immunogenicity of the quadrivalent vaccine, comprising mRNAs encoding gB2, gC2, gD2, and gE2, was evaluated using two different LNP formulation strategies: a co-formulated version, in which all antigen mRNAs were encapsulated in a single LNP, and an admixed version, in which each mRNA was encapsulated separately in individual LNPs (**Figure 2A**). Physicochemical characterization of the LNPs revealed that all four mRNAs had a high encapsulation efficiency (EE%) of approximately 90%. Both single and co-formulated LNPs exhibited a uniform size distribution, with Z-average diameters ranging from 86 to 117 nm, a range considered optimal for mRNA vaccine delivery (**Supplementary Figure S2**). Mice were immunized with 10 μg of each mRNA at a two-week interval, and sera were collected at 1 and 2 weeks post-second immunization (wpi) to measure total IgG and neutralizing antibody titers (**Figure 2B**). Both formulations induced substantial levels of HSV-2-specific IgG and neutralizing antibodies as early as 1 wpi, which further increased by week 2. Importantly, no significant differences in antibody titers were detected between the two formulation strategies (**Figures 2C, D**), indicating that the quadrivalent mRNA vaccine elicited comparable humoral immune responses, regardless of the LNP formulation method.

### 3.3 Protective efficacy of quadrivalent mRNA vaccines was compared between co-formulated and admixed LNP delivery methods

**Figure 3:**
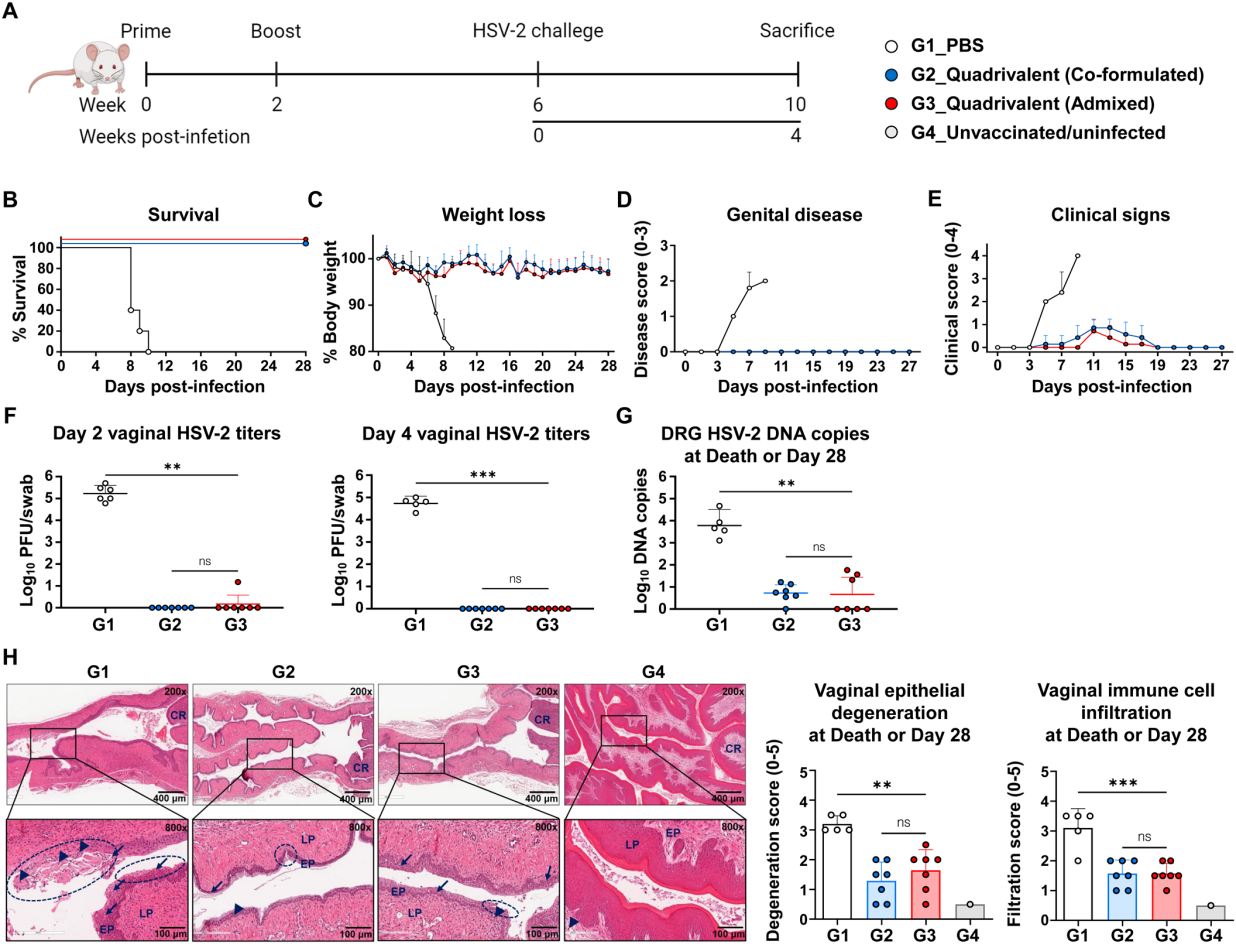
Comparison of protective efficacy between co-formulated and admixed quadrivalent mRNA vaccines. Four weeks after the second immunization, mice were intravaginally challenged with 1×10^5^ PFU of HSV-2 strain MS. **(A)** Immunization groups and experimental design for the viral challenge. **(B–E)** Survival **(B)** and weight loss **(C)** were monitored daily, and genital disease **(D)** and clinical signs **(E)** were scored every alternate day for 28 days. **(F)** HSV-2 titers on days 2 and 4 post-infection were determined using a plaque assay. **(G, H)** DRG and vaginal tissue were harvested at death or study endpoint. HSV-2 DNA copies in the DRG **(G)** were quantified by real-time PCR. H&E-stained vaginal sections **(H)** showing the epithelial layer (EP), lamina propria (LP), cervical region (CR), infected (necrotic) cells (arrows), immune cell infiltration (arrowheads), and degeneration of stratified squamous epithelium (circles). The challenge experiment was conducted using six to seven vaccinated mice in each group (G1, n = 6; G2, n = 7; G3, n = 7). On day 2 post-infection, one mouse in G1 died during anesthesia for vaginal swab collection, reducing the number of mice in G1 from day 4 onward (G1, n = 5). Unvaccinated and uninfected mice served as controls for the normal vaginal tissue (G4, n = 1). *P-*values were calculated using the Kruskal-Wallis test with Dunn’s multiple comparisons test **(F, G)** and one-way ANOVA with Tukey’s multiple comparisons test **(H)**. **p < 0.01, ***p < 0.001, ns, not significant. Error bars represent standard deviations of the means **(C–E, H)** and 95% confidence intervals of the geometric means **(F, G)**. PFU, plaque-forming units; DRG, dorsal root ganglia; H&E, hematoxylin and eosin.

Four weeks after completing the two-dose immunization regimen, mice were intravaginally challenged with 1×10^5^ PFU of HSV-2 strain MS. Survival, weight loss, genital disease, and clinical signs were monitored over 28 days (**Figure 3A**). Vaginal swabs were collected on days 2 and 4 post-infection to determine viral titers, and the dorsal root ganglia (DRG) and vaginal tissues were harvested at the time of death or the study endpoint for HSV-2 DNA quantification and histopathological analysis. Uninfected and unvaccinated mice were used as baseline controls for histological comparison. By day 10, all the mice in the PBS group had succumbed, resulting in a 0% survival rate. All mice immunized with either the co-formulated or admixed quadrivalent mRNA vaccine survived until the study endpoint (**Figure 3B**). Vaccinated mice did not show severe weight loss (**Figure 3C**) or genital disease (**Figure 3D**). The PBS group, however, developed genital disease by day 5 post-infection (**Figure 3D**), followed by rapid weight loss (**Figure 3C**). Mild clinical signs, such as slight fur ruffling, were observed in some vaccinated mice between days 5 and 17 post-infection (**Figure 3E**). On days 2 and 4 post-infection, the PBS group exhibited active viral replication at the infection site, with vaginal viral titers exceeding the geometric mean of 10^4^ PFU/swab. No detectable virus was observed on day 4 in either quadrivalent mRNA vaccine group, indicating early suppression of HSV-2 infection (**Figure 3F**). Furthermore, HSV-2 DNA was detected in the DRG of all PBS-treated mice (8–10 days post-infection, the time of death), with viral loads exceeding 10^3^ copies. Unlike the PBS group, the vaccinated mice exhibited a markedly lower latent viral burden by day 28, with geometric mean levels below 10 copies. Specifically, viral DNA was detected in six of the seven mice in the co-formulated group (4–16 copies) and in three of the seven mice in the admixed group (21–58 copies), but this difference was not statistically significant (p > 0.05) (**Figure 3G**). These findings demonstrated that the quadrivalent mRNA vaccine markedly reduced HSV-2 latency in the DRG.

Histopathological analysis of the vaginal tissues revealed severe pathological changes in the PBS group (8–10 days post-infection, the time of death), including epithelial erosion, atrophic necrosis, vacuolation, and atrophic degeneration. These lesions were markedly reduced in both the vaccine groups at the study endpoint (4 weeks post-infection) (**Figure 3H**). The PBS group also showed extensive immune cell infiltration, suggesting tissue damage (**Figure 3H**). In contrast, the vaccine groups exhibited milder but persistent immune cell infiltration, possibly reflecting ongoing tissue repair (19–22), residual responses to viral antigens (23–25), or immune cells preventing the reactivation of HSV-2 (26–30). Both quadrivalent mRNA vaccines, whether co-formulated or admixed, effectively suppressed viral replication, prevented disease, and reduced tissue damage and latent infection, with no significant difference in efficacy.

### 3.4 Quadrivalent mRNA vaccine elicited dose-dependent immune responses

**Figure 4:**
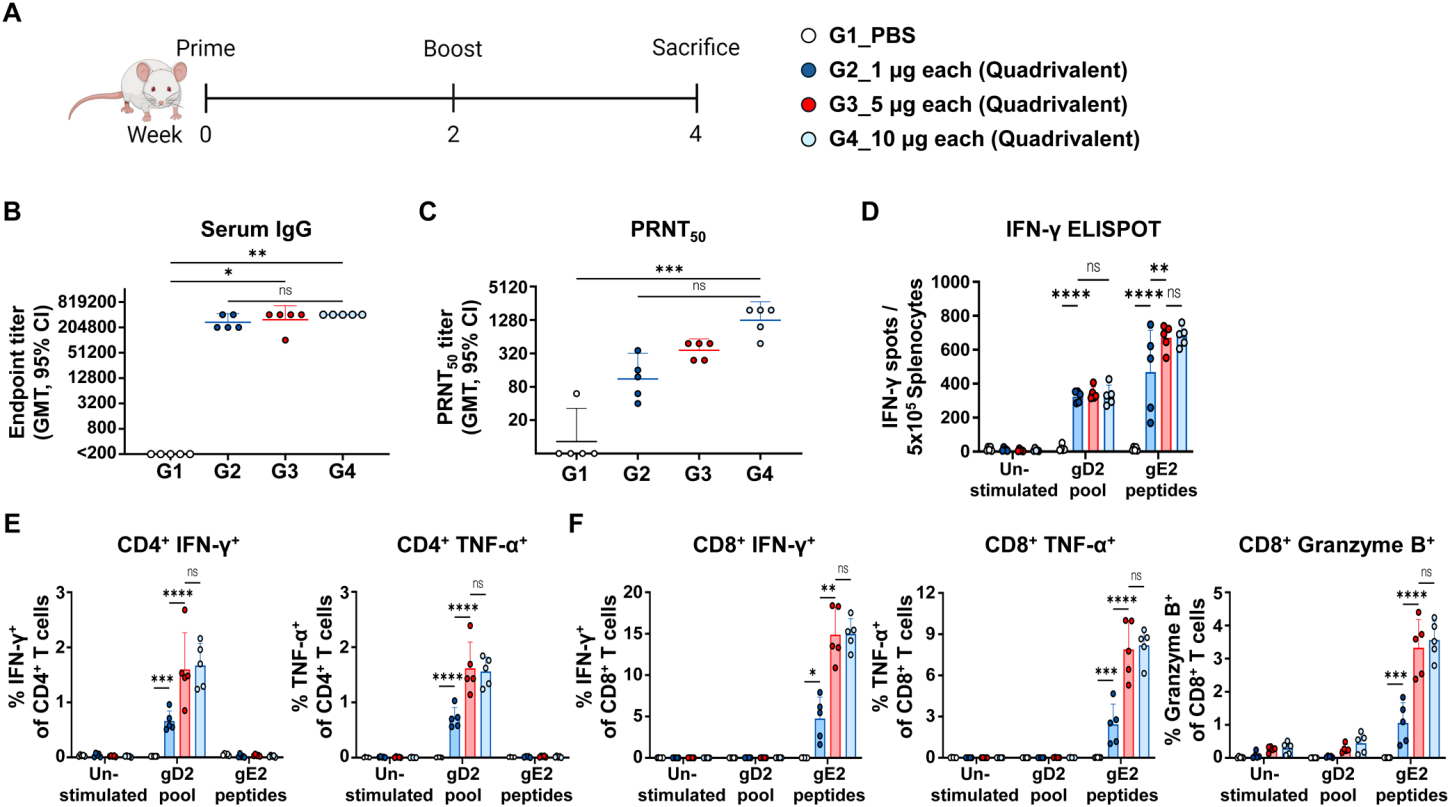
Immunogenicity based on the dose of quadrivalent mRNA vaccine. Mice were immunized twice intramuscularly with PBS or 1 μg, 5 μg, or 10 μg of co-formulated quadrivalent mRNA vaccine consisting of gB2, gC2, gD2, and gE2 mRNA. The indicated dose refers to the amount of mRNA produced per antigen. **(A)** Immunization groups and schedule. **(B, C)** Serum IgG titers **(B)** and PRNT_50_ neutralizing antibody titers **(C)** were measured using ELISA and PRNT, respectively. IgG titers: 1 μg dose *vs*. PBS (*p* = 0.1263); 5 μg dose *vs*. PBS (*p* = 0.0153); 10 μg dose *vs*. PBS (*p* = 0.0023). PRNT_50_ titers: 1 μg dose *vs*. PBS (*p* > 0.9999); 5 μg dose *vs*. PBS (*p* = 0.0574); 10 μg dose *vs*. PBS (*p* = 0.0006). **(D)** An IFN-γ ELISPOT assay was performed on splenocytes. **(E, F)** CD4^+^ T cells producing IFN-γ or TNF-α **(E)** and CD8^+^ T cells producing IFN-γ, TNF-α, or Granzyme B **(F)** in splenocytes were analyzed using ICS/flow cytometry. Splenocytes were stimulated with the gD2 peptide pool or the gE2 peptides before the ELISPOT and ICS/flow cytometry assays. Experiments involved five mice per group (n = 5/group). *P-*values were calculated using the Kruskal-Wallis test with Dunn’s multiple comparisons test **(B, C)** and two-way ANOVA with Tukey’s multiple comparisons test **(D–F)**. *p < 0.05, **p < 0.01, ***p < 0.001, ****p < 0.0001, ns, not significant. Error bars represent 95% confidence intervals of the geometric means **(B, C)** and standard deviations of the means **(D–F)**. GMT, geometric mean titer.

To investigate the dose-dependent immune response elicited by the quadrivalent mRNA vaccine, co-formulations containing 1 μg, 5 μg, or 10 μg of each antigen were administered to mice in two doses over two weeks. Antibody and cellular immune responses were assessed two weeks after the second dose (**Figure 4A**). Serum anti-HSV-2 IgG levels showed that the 1 μg dose generated substantially higher titers than the PBS control, whereas IgG titers in the 1 μg group were not significantly different from those in the 5 μg or 10 μg groups (**Figure 4B**). Neutralizing antibody titers increased with dose, with GMTs of 111, 364, and 1,267 in the 1 μg, 5 μg, and 10 μg doses, respectively. Although neutralizing antibody titers appeared to increase in a dose-dependent manner from 1 μg to 10 μg, no statistical significance was observed between the vaccinated groups (**Figure 4C**).

The cellular immune response was measured using the gD2 and gE2 peptides to stimulate CD4^+^ and CD8^+^ T cell responses, respectively. In IFN-γ ELISPOT assay, the 1 µg dose enhanced cellular responses, with a further increase observed at the 5 µg dose, but no additional enhancement detected at the 10 µg dose (**Figure 4D**). Consistent with the ELISPOT results, a dose-dependent trend was observed in CD4^+^ T cells producing IFN-γ or TNF-α (**Figure 4E**), as well as in CD8^+^ T cells producing IFN-γ, TNF-α, or Granzyme B (**Figure 4F**). Similarly, TEM and SLEC subsets among total CD8^+^ T cells increased at the 5 μg dose and showed no further increase at 10 μg (**Supplementary Figure S3**). The gating strategy and representative flow plots for cytokine-producing CD4^+^/CD8^+^ T cells and CD8^+^ SLEC/TEM subsets are shown in **Supplementary Figure S4**. Overall, the strongest neutralizing antibody response was achieved at the 10 μg dose, while cellular immunity peaked at 5 μg, indicating that this dose was sufficient to elicit maximal cellular immunity.

### 3.5 Quadrivalent mRNA vaccine elicited long-lasting immune responses and provided durable protection against infection

**Figure 5:**
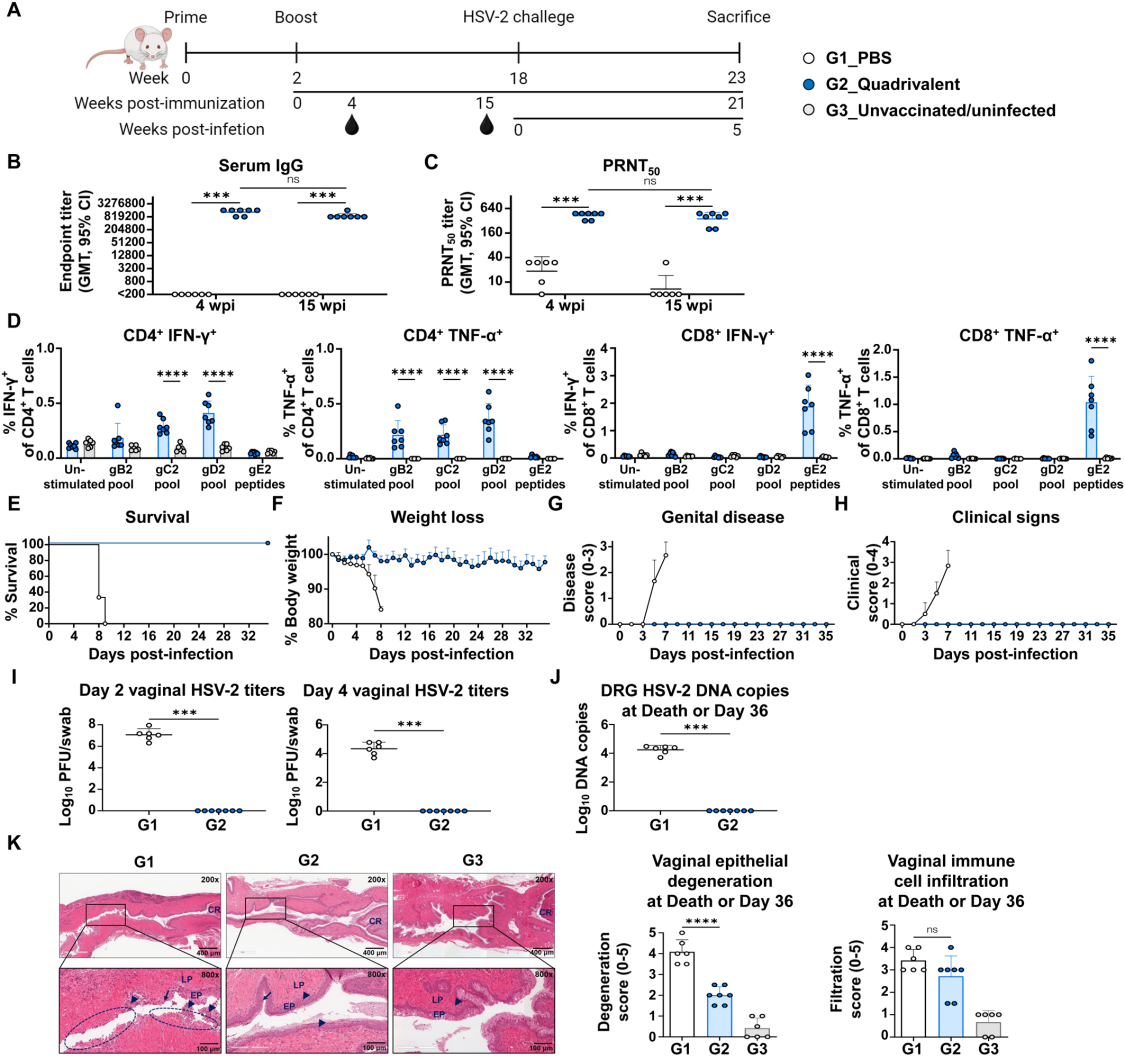
Evaluation of long-term immune responses and protective efficacy of quadrivalent mRNA vaccine. Mice were intravaginally challenged with 1×10^5^ PFU of HSV-2 strain MS 16 weeks after two intramuscular immunizations with PBS or co-formulated quadrivalent mRNA vaccine (10 μg of each gB2, gC2, gD2, and gE2 mRNA). **(A)** Immunization groups and experimental design for the viral challenge. **(B, C)** Serum IgG titers **(B)** and PRNT_50_ neutralizing antibody titers **(C)** at weeks 4 and 15 post-second immunization. **(D)** IFN-γ- or TNF-α-producing splenic CD4^+^ and CD8^+^ T cells were assessed after peptide stimulation at the study endpoint. **(E–H)** Survival **(E)** and weight loss **(F)** were monitored daily, and genital disease **(G)** and clinical signs **(H)** were scored every alternate day for 36 days. **(I)** HSV-2 titers on days 2 and 4 post-infection. **(J, K)** DRG and vaginal tissue were harvested at death or study endpoint. HSV-2 DNA copies in the DRG **(J)** and H&E-stained vaginal sections showing epithelial degeneration and immune cell infiltration **(K)**. Experiments involved six to seven mice per group (G1, n = 6; G2, n = 7). Unvaccinated and uninfected mice served as controls for baseline T cell levels and normal vaginal tissues (G3, n = 6). *P-*values were calculated using the two-tailed Mann-Whitney U test **(B, C, I, J)**, two-tailed Wilcoxon signed-rank test for within-group comparison **(B, C)**, two-way ANOVA with Sidak’s multiple comparisons test **(D)**, and one-way ANOVA with Tukey’s multiple comparisons test **(K)**. ***p < 0.001, ****p < 0.0001, ns, not significant. Error bars represent 95% confidence intervals of the geometric means **(B, C, I, J)** and standard deviations of the means **(D, F–H, K)**. GMT, geometric mean titer; wpi, weeks post-second immunization.

To assess the long-term immune-protective efficacy of the quadrivalent mRNA vaccine, mice were challenged 16 weeks after the second immunization (**Figure 5A**). As shown in **Figures 5B, C**, anti-HSV-2 IgG levels and neutralizing antibody titers measured at week 15 post-second immunization remained high, with only a minimal decline from week 4 (IgG GMT: 1,344,037 to 904,470; PRNT_50_ GMT: 427 to 355), indicating durable humoral responses that likely contributed to early and effective control of viral replication. In addition to humoral immunity, splenocytes collected at the study endpoint (21 wpi) showed persistent T cell responses specific to all four vaccine antigens (gB2, gC2, gD2, and gE2), demonstrating the longevity of cellular immunity (**Figure 5D**).

All mice in the PBS group succumbed to the infection by day 9 after challenge, whereas all vaccinated mice survived until day 36 (5 weeks post-infection), the study endpoint (21 wpi) (**Figure 5E**). Vaccinated mice exhibited no significant weight loss (**Figure 5F****),** genital disease (**Figure 5G**), or clinical signs (**Figure 5H**) during the observation period. Notably, even during the early phase of infection (days 2–4 after challenge), when viral replication is typically active (16, 31, 32), vaccinated mice maintained a stable body weight, suggesting early viral control. This was supported by vaginal viral titers measured on day 2 post-infection, which revealed high viral loads in PBS-treated mice (GMT, 1.2×10^7^), while no virus was detected in the vaccinated mice, indicating potent suppression of early viral replication (**Figure 5I**). Quantification of HSV-2 DNA in the DRG on day 36 (5 weeks post-infection) revealed that the viral genome was undetectable in vaccinated mice, suggesting that the vaccine effectively blocked neuroinvasion and the establishment of latency (**Figure 5J**). However, gD2 mRNA alone did not fully prevent viral replication or HSV-2 DRG infection (**Supplementary Figure S5**). When comparing protective efficacy with the trivalent vaccine, a difference was observed in vaginal viral titers at 2 days post-infection. Although this difference was not statistically significant, the quadrivalent vaccine showed undetectable virus, whereas virus was detected in 29% (2 of 7) of mice in the trivalent vaccine group (**Supplementary Figure S6****).** Histological analysis confirmed the protective effects of the vaccination. PBS-treated mice (G1) exhibited severe epithelial degeneration and prominent infiltration of immune cells by days 8–9 post-infection, whereas vaccinated mice showed milder epithelial disruption and immune cell infiltration on day 36 (5 weeks post-infection). The presence of macrophages, neutrophils, and lymphocytes in vaginal tissues at this late time point may reflect ongoing immune surveillance and tissue remodeling (**Figure 5K**).

Collectively, these results indicated that the quadrivalent mRNA vaccine induced durable and comprehensive immune responses, providing full protection against HSV-2 by preventing genital herpes, suppressing viral replication, and blocking latent infections.

## 4 Discussion

Prophylactic vaccination is the most effective and practical strategy for preventing new genital infections caused by HSV-2. Previous studies have shown that gC2 and gE2, when administered individually, confer limited protection against HSV-2 genital herpes and DRG infections compared to gD2. However, their inclusion along with gD2 enhances overall vaccine efficacy (8, 16, 33). A recent study using a trivalent mRNA vaccine encoding gC2, gD2, and gE2 showed significantly improved T cell and antibody responses, as well as protection against subclinical infection, compared to the protein subunit formulation (9). Moreover, the mRNA platform elicits a more robust and durable B cell immune response, resulting in sustained protection against mortality, genital lesions, and latent infection (34). These findings highlight the immunological advantages of endogenous antigen expression through mRNA vaccines, which may underlie their superior protective efficacy compared with traditional protein-based approaches.

To broaden immunogenic diversity and protective efficacy, we incorporated gB2, a highly conserved glycoprotein essential for HSV-2 entry and cell-to-cell spread (35–37), into a quadrivalent mRNA vaccine along with gC2, gD2, and gE2. gB2 also serves as a major target for Fc-mediated antibody responses even in the absence of neutralization (38). In our quadrivalent formulation, the inclusion of gB2 likely broadened the antibody repertoire and enhanced cross-functional immunity. Immunogenicity profiling of the quadrivalent formulation revealed that gB2 mRNA induced robust antibody production as well as both CD4^+^ and CD8^+^ T cell responses, thereby expanding the overall immune repertoire. This antigenic combination elicited a coordinated immune response characterized by high titers of IgG1, IgG2a, and neutralizing antibodies, Th2-associated humoral responses, and strong Th1 and Tc1 cellular responses. Importantly, Th1-biased antibodies and Th1 cytokines may contribute to preventing reactivation. Th1-biased antibodies (mouse IgG2a) possess stronger Fc-mediated effector functions than Th2-biased antibodies (IgG1) (39, 40), promoting rapid clearance of infected epithelial cells via antibody-dependent cell-mediated cytotoxicity and complement-dependent cytotoxicity. These functions act synergistically with the cytotoxic CD8^+^ TEM, which, upon viral reactivation, migrate to infection sites (41), secrete IFN-γ and TNF-α, and eliminate infected cells through granzyme B-mediated cytotoxicity. IFN-γ and TNF-α activate antiviral pathways that suppress HSV replication, partly through the induction of indoleamine 2,3-dioxygenase, which depletes tryptophan required for viral growth (42), and the upregulation of interferon-γ-inducible protein 16, which suppresses the expression of lytic gene ICP0 (43). Collectively, these mechanisms likely underpin the latency suppression observed in our model.

Of the four glycoproteins, gE2, unlike gB2, gC2, and gD2, elicited only low levels of anti-HSV-2 lysate IgG and neutralizing antibodies. This weak antibody response may result from several factors. First, gC2 mediates cell attachment, while gB2 and gD2 are essential for cell entry and fusion; however, gE2 does not directly participate in the initial HSV-2 infection process (5, 6). Therefore, antibodies generated against gE2 may be ineffective at neutralizing the virus. Second, the gE2–gI2 complex binds to the Fc region of IgG as an immune evasion strategy (33, 44). This Fc binding may partially mask gE2 epitopes, hindering the binding of gE2-specific antibodies. Finally, the comparatively weak Th2 response observed in this study, together with the weak activation signal through the B cell receptor due to the non-secreted form of the antigen, which contains a transmembrane domain, may have acted synergistically to limit B cell activation and thus antibody production. Nonetheless, the primary role of gE2 mRNA in the quadrivalent HSV-2 vaccine is to serve as an antigen that induces CD8^+^ T cell responses rather than neutralizing antibodies.

Animals immunized with the quadrivalent mRNA vaccine demonstrated complete protection against genital herpes and DRG infections up to 16 weeks post-vaccination, accompanied by rapid suppression of vaginal viral replication. However, gD2 mRNA alone offered only partial protection, underscoring the additive value of targeting multiple non-redundant viral components (**Supplementary Figure S5**). Moreover, this antigenic combination maintained durable antibody titers and T cell responses, with CD4^+^ T cells recognizing gB2, gC2, and gD2, and CD8^+^ T cell responses restricted mainly to gE2. Although the overall magnitudes decreased over time, these responses persisted throughout the memory phase. In our quadrivalent vaccine, durable humoral and cellular responses—including persistent IgG titers, balanced Th1/Th2 and Tc1 responses, and reduced latent HSV-2 DNA—indicate the induction of long-lived immune memory. Because latent HSV-2 infection and reactivation occur over much longer periods in humans (45), evaluating protection for 16 weeks in mice provides a biologically meaningful surrogate for multi-year immune maintenance in humans (46, 47). Hence, persistence of multifunctional T cells and memory B cells will be essential for durable efficacy, and long-term studies will be required to confirm protection beyond 16 weeks. These findings underscore the benefits of targeting multiple non-redundant viral components using a multivalent mRNA strategy to achieve sustained and comprehensive protection against HSV-2. While the overall protection was comparable between the trivalent and quadrivalent mRNA vaccines, the undetectable early vaginal viral titers observed only in the quadrivalent group could suggest a marginal advantage in immediate viral control (**Supplementary Figure S6****).**

In a dose-ranging study, two doses of 10 μg per antigen of the quadrivalent mRNA vaccine achieved adequate protection against HSV-2 infection in mice. Notably, the 5 μg dose was sufficient to elicit maximal cellular immune responses, while neutralizing antibody titers continued to increase with antigen dose up to 10 μg. These results suggest that the optimal dose may lie between 5 μg and 10 μg. Further studies evaluating long-term immunogenicity and protective efficacy in relevant disease models are warranted to define the minimal effective dose and guide translational development.

Although dose optimization is essential for balancing immunogenicity and efficacy, the formulation strategy is another critical aspect of multivalent mRNA vaccine development. A key consideration is whether to co-encapsulate all antigen-encoding mRNAs in a single LNP (co-formulation) or formulate them separately (admixed formulation). The formulation approach can affect not only the immune performance but also the manufacturing efficiency and quality control. Here, the admixed and co-formulated quadrivalent mRNA vaccines produced comparable antibody responses, neutralizing antibody responses, and protective efficacy, indicating that immunological outcomes were not affected by the formulation method. Furthermore, activation of CD4^+^ and CD8^+^ T cells by gD2 and gE2, respectively, indicates that the co-formulation approach effectively induces immune responses to all four antigens. gB2, gC2, gD2, and gE2 mRNAs vary in length (1,608–3,135 nucleotides) and ensemble free energy (−572.34 to −1,402.42 kcal/mol) but share similar GC content (57–63%) and a minimum free energy structure frequency of 0%, indicating that they exist as a diverse ensemble of flexible structures (48). When formulated individually, each mRNA showed a comparable EE% of approximately 90% (**Supplementary Figure S2A**), suggesting that the LNP system is broadly effective across this range of mRNA lengths and secondary structural stabilities (49). The monodispersed particle size (PDI < 0.2), ranging from 86 to 117 nm in single and co-formulated LNPs, further supports the broad efficacy of the LNP system (**Supplementary Figure S2B****).** Consistent manufacturing was confirmed, with a coefficient of variation of 5.73% in LNP size across the four co-formulated batches. In a scalable GMP manufacturing process, co-formulation greatly reduces the quality control (QC) burden, as it represents a single mRNA–LNP drug substance (DS). This is because an admixed formulation requires individual QC for each DS, whereas in a co-formulation, all tests are performed on the single DS. Our study supports the practical application of this co-formulation strategy, demonstrating that the co-formulated quadrivalent vaccine elicited distinct immune responses to all four antigens. These results suggest that the formulation strategy can be optimized based on practical considerations, such as manufacturability, quality control, and cost, without compromising vaccine efficacy. Nonetheless, while co-formulation is preferred due to its efficiency in manufacturing processes, further studies are needed to confirm the EE% of each mRNA within the co-formulated LNPs and to validate whether co-formulation supports efficient delivery and balanced expression across all encoded antigens.

Overall, the quadrivalent mRNA vaccine incorporating gB2, gC2, gD2, and gE2 provided strong protection against vaginal viral replication, genital herpes, and latent infection, while inducing durable and broad humoral and cellular immune responses. These findings support its potential as a promising candidate for preventing HSV-2 infection. Furthermore, the quadrivalent mRNA vaccine developed in this study not only induced strong antibody responses but also elicited robust CD4^+^ and CD8^+^ T cell responses, suggesting that this mRNA vaccine may have potential not only as a preventive vaccine but also as a therapeutic vaccine. However, this study was unable to assess viral reactivation or recurrent shedding because spontaneous HSV-2 reactivation did not occur in the mice (50, 51). Future studies using reactivation-competent models are crucial for a comprehensive evaluation of the long-term efficacy of these vaccines.

## Data Availability

The original contributions presented in the study are included in the article/**Supplementary Material**. Further inquiries can be directed to the corresponding authors.
