# Supplementary material for: Design and immunogenicity of a quadrivalent mRNA vaccine targeting HSV-2 with comparative evaluation of co-formulated and admixed formulations
Source: Front Immunol. 2025 Dec 5;16:1712691. doi: 10.3389/fimmu.2025.1712691 (PMC12714894; doi:10.3389/fimmu.2025.1712691)
Supplement: Supplementary file 1 [file DataSheet1.docx]

Supplementary Material

**Design and Immunogenicity of a Quadrivalent mRNA Vaccine Targeting HSV-2 with Comparative Evaluation of Co-formulated and Admixed Formulations**

**Youngran Cho****^1,2,^****^†^, Chanwoo Lee^3,†^, Sang-In Park^5^, Yeongjun Kim^3^, Yu-Sun Lee^1^, Seonghyun Lee^1,2^, Subin Yoon^1,2^, Gahyun Roh^1^, Dahyeon Ha^1,2^, Ayoung Oh^1,2^, Kyusang Cho^1^, Jisun Lee^1^, Hyo-Jung Park^1,2^, Hye-Ra Lee^3,4,^*, Jae-Hwan Nam^1,2,^***

^1^Department of Medical and Biological Sciences, The Catholic University of Korea, Bucheon, Gyeonggi-do, Republic of Korea.

^2^BK21 Four Department of Biotechnology, The Catholic University of Korea, Bucheon, Gyeonggi-do, Republic of Korea.

^3^Department of Biotechnology & Bioinformatics, College of Science and Technology, Korea University Sejong Campus, Sejong City, Republic of Korea.

^4^Department of Lab Medicine, College of Medicine, Korea University, Seoul, 02841, Republic of Korea.

^5^Department of Biomedical Laboratory Science, Daegu Haany University, Gyeongsan, Gyeongsangbuk-do, Republic of Korea.

**^†^**These authors contributed equally to this work and share first authorship.

*** Correspondence:**Hye-Ra Lee: leehr@korea.ac.kr; Jae-Hwan Nam: [jhnam@catholic.ac.kr](mailto:jhnam@catholic.ac.kr)

# Supplementary Figures


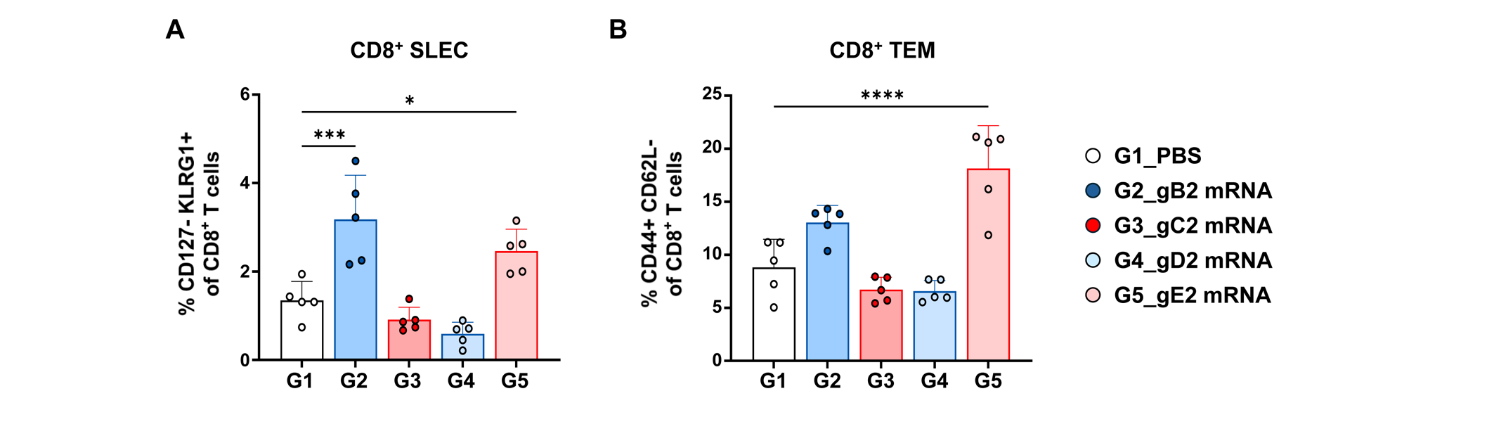


**Supplementary Figure S1. Analysis of SLEC and TEM subsets within CD8^+^ T cells following immunization with gB2, gC2, gD2, or gE2 mRNA.** Mice were immunized twice at a two-week interval intramuscularly with PBS or 10 μg of gB2, gC2, gD2, or gE2 mRNA. (A, B) CD8^+^ SLEC (A) and CD8^+^ TEM (B) in splenocytes were analyzed by flow cytometry 2 weeks after the second immunization. Each group contained five mice (n = 5/group). *P-*values were calculated using one-way ANOVA with Tukey’s multiple comparisons test. **p* < 0.05, ****p* < 0.001, *****p* < 0.0001. Error bars represent standard deviations of the means. SLEC: short-lived effector cells; TEM: effector memory T cells.

**
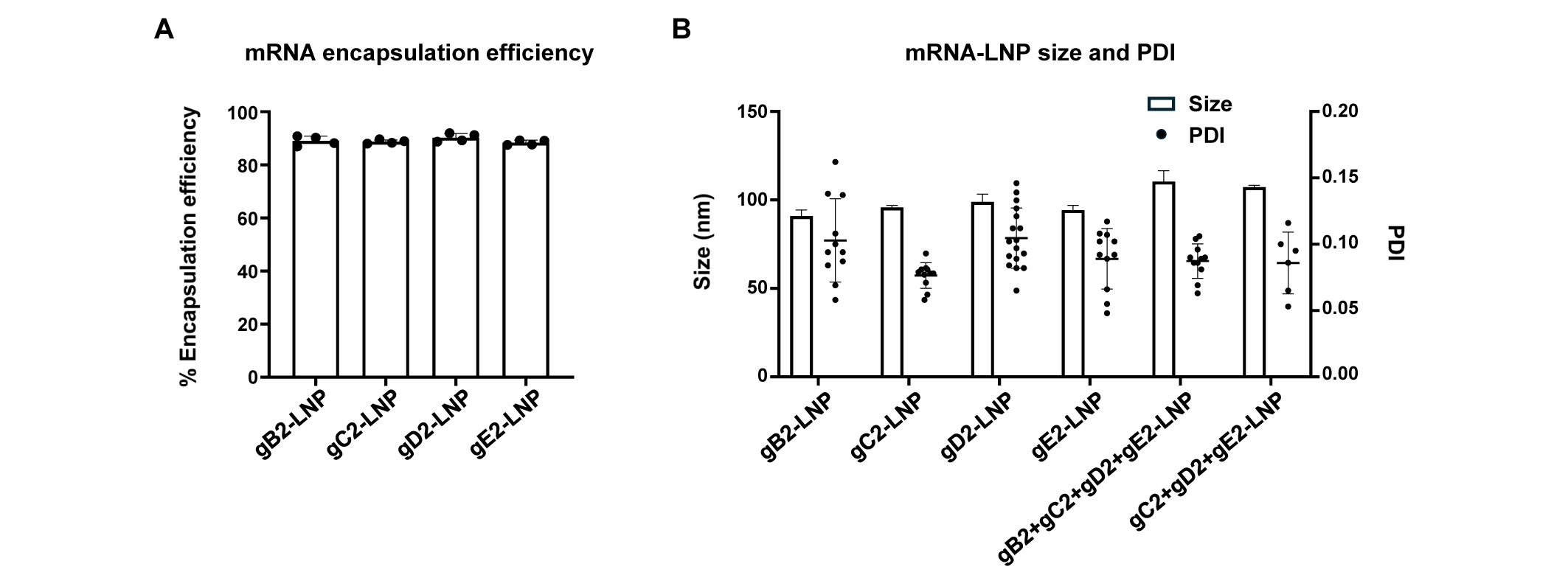
**

Supplementary Figure S2. Encapsulation efficiency of gB2, gC2, gD2, and gE2 mRNAs and DLS characterization of the mRNA-loaded LNPs. (A) Encapsulation efficiency (EE%) of each mRNA was quantified by RiboGreen assay. EE% was calculated as (Total mRNA – Unencapsulated mRNA) / Total mRNA × 100. Data were obtained from two batches, each measured in duplicate (n = 4). (B) Z-average particle size and PDI of the mRNA-loaded LNPs were measured using DLS. Data were obtained from all production batches (two to six batches) used in the study, with each measured in duplicate or triplicate (gB2-LNP, n = 11; gC2-LNP, n = 11; gD2-LNP, n = 17; gE2-LNP, n = 11; gB2+gC2+gD2+gE2-LNP, n = 11; gC2+gD2+gE2-LNP, n=6). Error bars represent standard deviations of the means. DLS: dynamic light scattering; PDI: polydispersity index; LNP: lipid nanoparticle.


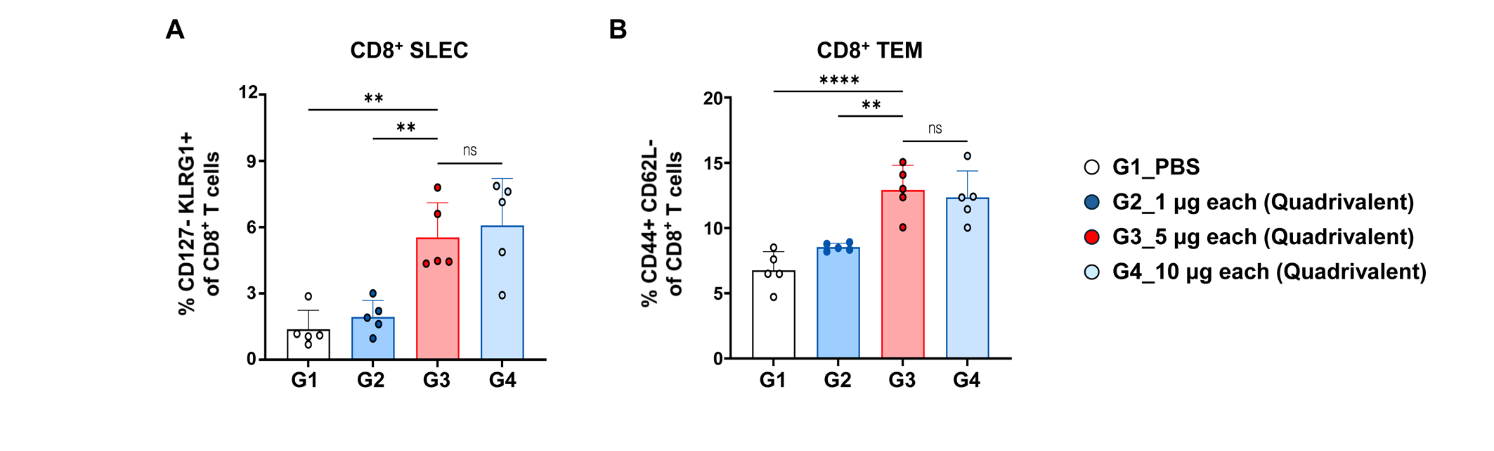


**Supplementary Figure S3. Analysis of SLEC and TEM subsets within CD8^+^ T cells following immunization with different doses of quadrivalent mRNA vaccine**. Mice were immunized twice at a two-week interval intramuscularly with PBS or 1 μg, 5 μg, or 10 μg of co-formulated quadrivalent mRNA vaccine consisting of gB2, gC2, gD2, and gE2 mRNAs. The indicated dose refers to the amount of mRNA produced per antigen. (A, B) CD8^+^ SLEC (A) and CD8^+^ TEM (B) in splenocytes were analyzed by flow cytometry 2 weeks after the second immunization. Each group contained five mice (n = 5/group). *P-*values were calculated using one-way ANOVA with Tukey’s multiple comparisons test. ***p* < 0.01, *****p* < 0.0001, ns, not significant. Error bars represent standard deviations of the means.


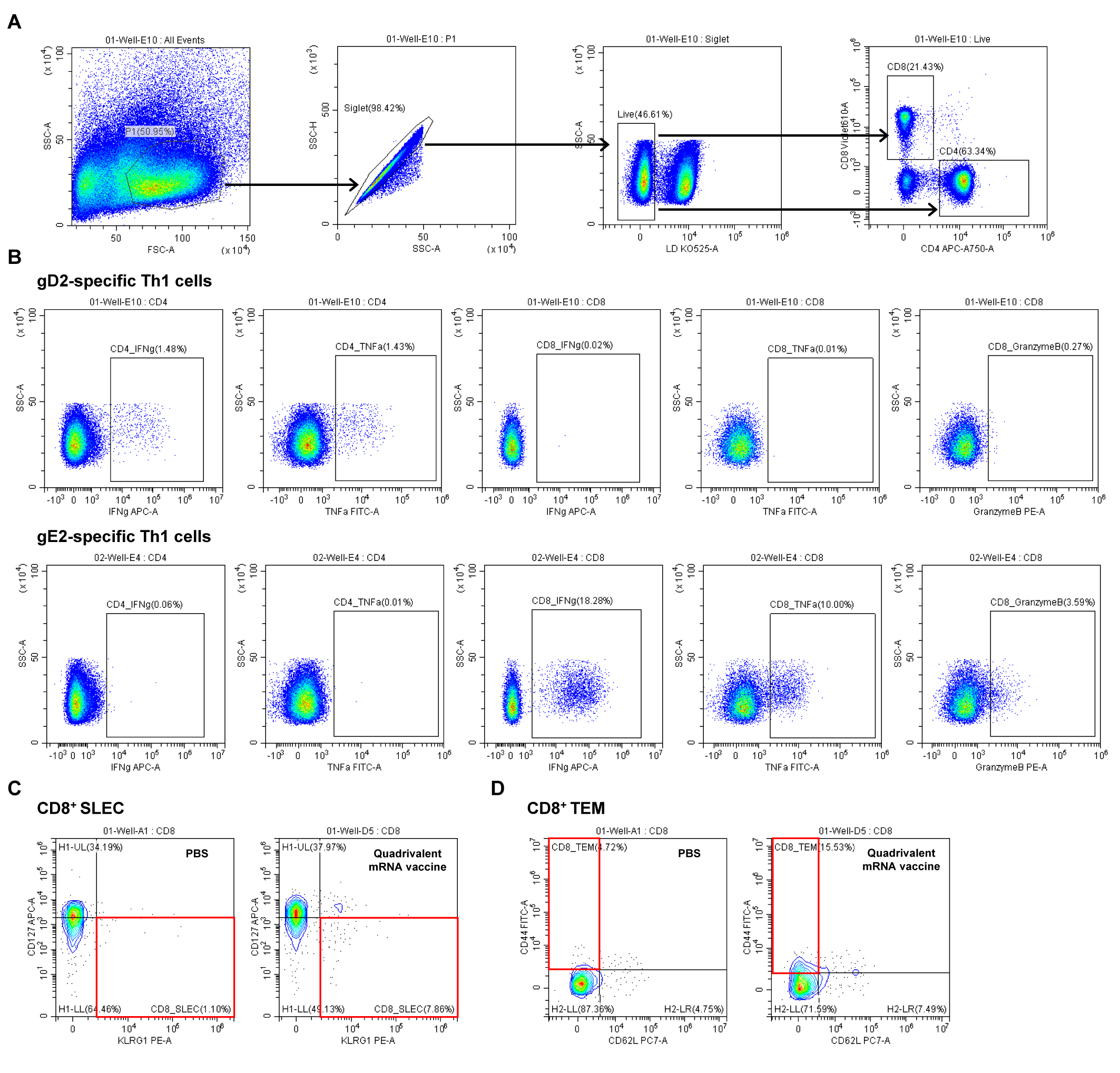


**Supplementary Figure S4. Gating strategies for flow cytometric analysis of antigen-specific cytokine-producing CD4^+^ and CD8^+^ T cells, and CD8^+^ SLEC/TEM subsets** (A) Gating strategy used to identify CD4^+^ and CD8^+^ T cells from splenocytes. (B) Representative flow plots showing CD4^+^ and CD8^+^ T cells producing Th1-type cytokines (IFN-γ, TNF-α) and cytotoxic molecule Granzyme B upon antigen stimulation. Corresponding data are shown in Figures 4E and 4F. (C) Representative flow plots showing CD8^+^ SLEC (CD127^-^ KLRG1^+^). Corresponding data are shown in Supplementary Figure 3A. (D) Representative flow plots showing CD8^+^ TEM (CD44^+^ CD62L^-^). Corresponding data are shown in Supplementary Figure 3B.


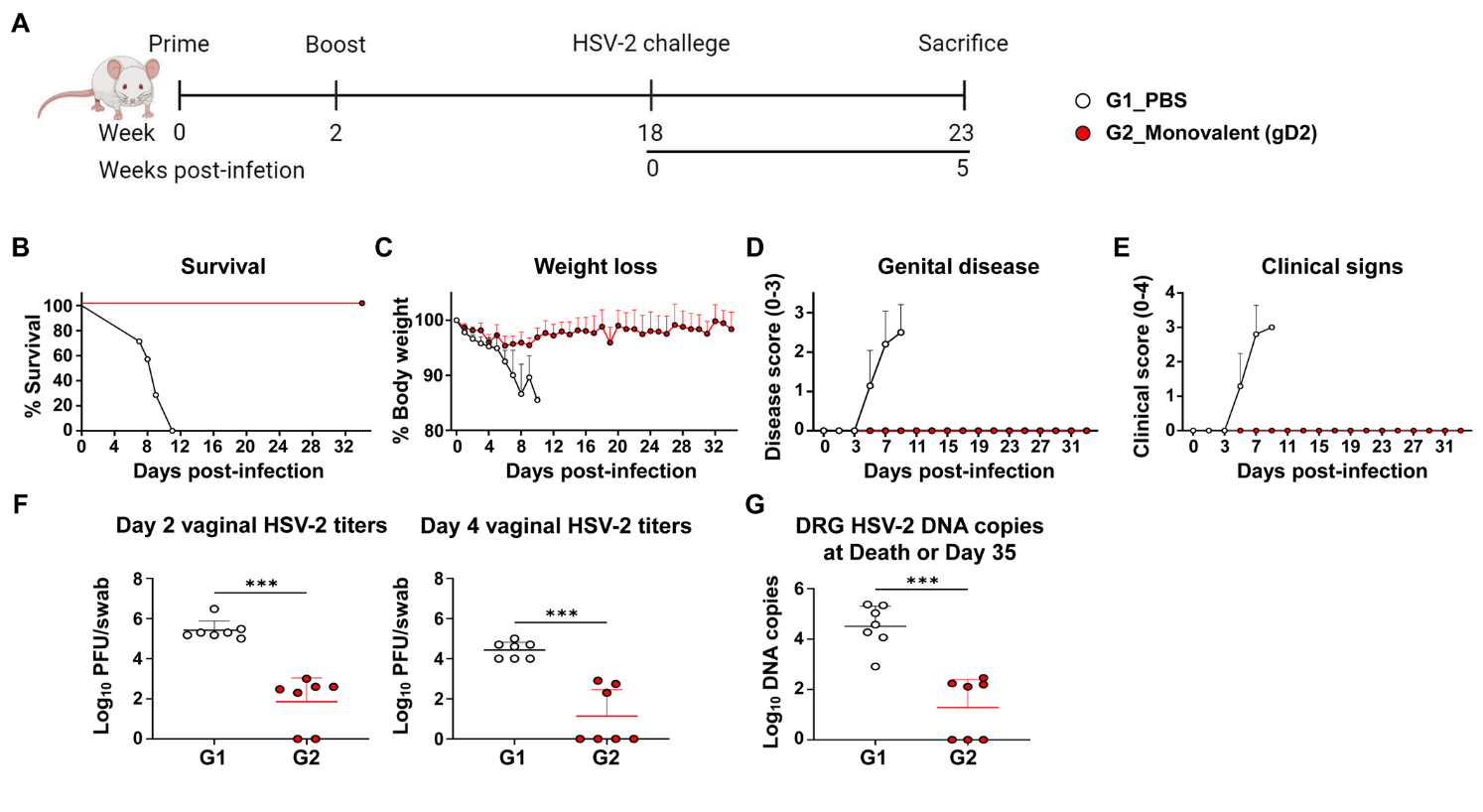


**Supplementary Figure S5. Evaluation of the long-term protective efficacy of the gD2 mRNA vaccine.** Mice were intravaginally challenged with 1×10^5^ PFU of HSV-2 strain MS 16 weeks after two intramuscular immunizations with PBS or 10 μg of gD2 mRNA vaccine. (A) Immunization groups and experimental design for the viral challenge. (B–E) Survival (B) and weight loss (C) were monitored daily, and genital disease (D) and clinical signs (E) were scored every alternate day for 35 days. (F) HSV-2 titers on days 2 and 4 post-infection were determined using a plaque assay. (G) HSV-2 DNA copies in the DRG were quantified by real-time PCR. DRG were harvested at death or study endpoint. Each group contained seven mice (n = 7/group). *P-*values were calculated using the two-tailed Mann-Whitney U test (F, G). ****p* < 0.001. Error bars represent standard deviations of the means (C–E) and 95% confidence intervals of the geometric means (F, G).


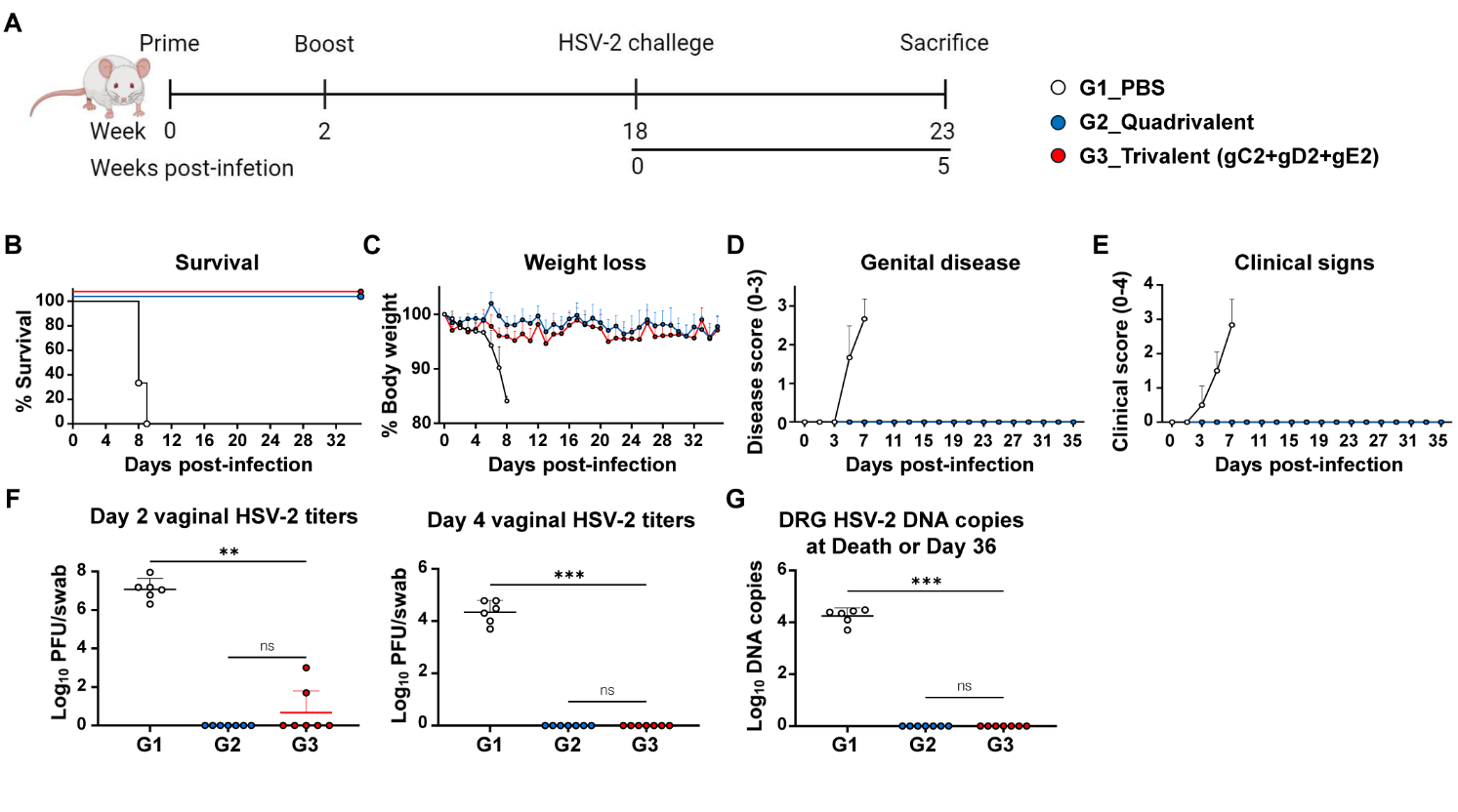


**Supplementary Figure S6. Comparison of protective efficacy between trivalent and quadrivalent mRNA vaccines.** Mice were intravaginally challenged with 1×10^5^ PFU of HSV-2 strain MS 16 weeks after two intramuscular immunizations with PBS, co-formulated quadrivalent, or co-formulated trivalent vaccine. 10 μg of mRNA per antigen was used for each group. (A) Immunization groups and experimental design for the viral challenge. (B–E) Survival (B) and weight loss (C) were monitored daily, and genital disease (D) and clinical signs (E) were scored every alternate day for 36 days. (F) HSV-2 titers on days 2 and 4 post-infection were determined using a plaque assay. (G) HSV-2 DNA copies in the DRG were quantified by real-time PCR. DRG were harvested at death or study endpoint. Experiments involved six to seven mice (G1, n = 6; G2, n = 7; G3, n=7). *P*-values were calculated using the Kruskal-Wallis test with Dunn’s multiple comparisons test (F, G). ***p* < 0.01, ****p* < 0.001, ns, not significant. Error bars represent standard deviations of the means (C–E) and 95% confidence intervals of the geometric means (F, G).
